# Predictors of Positive Surgical Margins after Robot-Assisted Partial Nephrectomy for Localized Renal Tumors: Insights from a Large Multicenter International Prospective Observational Project (The Surface-Intermediate-Base Margin Score Consortium)

**DOI:** 10.3390/jcm11071765

**Published:** 2022-03-23

**Authors:** Fabrizio Di Maida, Riccardo Campi, Brian R. Lane, Ottavio De Cobelli, Francesco Sanguedolce, Georgios Hatzichristodoulou, Alessandro Antonelli, Antonio Andrea Grosso, Sabrina Noyes, Oscar Rodriguez-Faba, Frank X. Keeley, Johan Langenhuijsen, Gennaro Musi, Tobias Klatte, Marco Roscigno, Bulent Akdogan, Maria Furlan, Claudio Simeone, Nihat Karakoyunlu, Martin Marszalek, Umberto Capitanio, Alessandro Volpe, Sabine Brookman-May, Jürgen E. Gschwend, Marc C. Smaldone, Robert G. Uzzo, Alexander Kutikov, Andrea Minervini, SIB International Consortium

**Affiliations:** 1 Unit of Oncologic Minimally-Invasive Urology and Andrology, Department of Experimental and Clinical Medicine, Careggi Hospital, 50134 Florence, Italy; fabridima90@gmail.com (F.D.M.); grossoantonioandrea@gmail.com (A.A.G.); 2 Unit of Urological Robotic Surgery and Renal Transplantation, Department of Experimental and Clinical Medicine, University of Florence, Careggi Hospital, 50134 Florence, Italy; riccardo.campi@gmail.com; 3 Department of Urology, Spectrum Health Medical Group, Grand Rapids, MI 49508, USA; brian.lane@spectrumhealth.org (B.R.L.); sabrina.noyes@spectrumhealth.org (S.N.); 4 Department of Urology, European Institute of Oncology (IEO), University of Milan, 20141 Milan, Italy; ottavio.decobelli@ieo.it (O.D.C.); gennaro.musi@ieo.it (G.M.); 5 Bristol Urological Institute, Southmead Hospital, Bristol BS10 5NB, UK; fsangue@hotmail.com (F.S.); francis.keeley@nbt.nhs.uk (F.X.K.); 6 Uro-Oncology Unit, Fundacio Puigvert, 08025 Barcelona, Spain; orodriguez@fundacio-puigvert.es; 7 Department of Urology, Rechts der Isar University Hospital, Technical University of Munich, 81675 Munich, Germany; georgios.hatzichristodoulou@gmail.com (G.H.); juergen.gschwend@tum.de (J.E.G.); 8 Department of Urology, Martha-Maria Hospital Nuremberg, 90491 Nurnberg, Germany; 9 Department of Urology, University of Brescia, 25121 Brescia, Italy; alessandro_antonelli@me.com (A.A.); mariachiara.furlan@gmail.com (M.F.); claudio.simeone@unibr.it (C.S.); 10 Department of Urology, University of Verona, Azienda Ospedaliera Universitaria Integrata Verona, 37126 Verona, Italy; 11 Department of Urology, Radboud University Nijmegen Medical Centre, 6525 GA Nijmegen, The Netherlands; hans.langenhuijsen@radboudumc.nl; 12 Department of Urology, Royal Bournemouth Hospital, Bournemouth BH7 7DW, UK; tobias.klatte@gmx.de; 13 Department of Urology, Medical University of Vienna, 1090 Vienna, Austria; 14 Department of Urology, ASST Papa Giovanni XXIII, 24127 Bergamo, Italy; roscigno.marco@gmail.com; 15 Department of Urology, School of Medicine, Hacettepe University, Ankara 06800, Turkey; blntakdogan@yahoo.com; 16 Department of Urology, Diskapi Yildirim Beyazit Training and Research Hospital, Ankara 06145, Turkey; niha.karakoyunlu@gmail.com; 17 Department of Urology and Andrology, Sozialmedizinishes Zentrum Ost-Donauspital, 1220 Vienna, Austria; martin.marszalek@wienkav.at; 18 Department of Urology, Graz Medical University, 8036 Graz, Austria; 19 Unit of Urology, Division of Experimental Oncology, Urological Research Institute (URI), IRCCS Ospedale San Raffaele, 20132 Milan, Italy; umbertocapitanio@gmail.com; 20 Department of Urology, Maggiore della Carità Hospital, University of Eastern Piedmont, 28100 Novara, Italy; foxal@tin.it; 21 Department of Urology, Campus Grosshadern, Ludwig-Maximilians University (LMU), 80539 Munich, Germany; sabine-brookman-amissah@web.de; 22 Janssen Pharma Research and Development, San Diego, CA 92121, USA; 23 Division of Urologic Oncology, Fox Chase Cancer Center, Philadelphia, PA 19111, USA; marc.smaldone@fccc.edu (M.C.S.); robert.uzzo@fccc.edu (R.G.U.); akutikov@gmail.com (A.K.)

**Keywords:** partial nephrectomy, positive surgical margins, renal tumor, robotics, SIB score

## Abstract

Background: To explore predictors of positive surgical margins (PSM) after robotic partial nephrectomy (PN) in a large multicenter international observational project, harnessing the Surface-Intermediate-Base (SIB) margin score to report the resection technique after PN in a standardized way. Methods: Data from consecutive patients with cT1-2N0M0 renal masses treated with PN from September 2014 to March 2015 at 16 tertiary referral centers and included in the SIB margin score International Consortium were prospectively collected. For the present study, only patients treated with robotic PN were included. Uni- and multivariable analysis were fitted to explore clinical and surgical predictors of PSMs after PN. Results: Overall, 289 patients were enrolled. Median (IQR) preoperative tumor size was 3.0 (2.3–4.2) cm and median (IQR) PADUA score was 8 (7–9). SIB scores of 0–2 (enucleation), 3–4 (enucleoresection) and 5 (resection) were reported in 53.3%, 27.3% and 19.4% of cases, respectively. A PSM was recorded in 18 (6.2%) patients. PSM rate was 4.5%, 11.4% and 3.6% in case of enucleation, enucleoresection and resection, respectively. Patients with PSMs had tumors with a higher rate of contact with the urinary collecting system (55.6% vs. 27.3%; *p* < 0.001) and a longer median warm ischemia time (22 vs. 16 min; *p* = 0.02) compared with patients with negative surgical margins, while no differences emerged between the two groups in terms of other tumor features (i.e., pathological diameter, PADUA score). In multivariable analysis, only enucleoresection (SIB score 3–4) versus enucleation (SIB score 0–2) was found to be an independent predictor of PSM at final pathology (HR: 2.68; 95% CI: 1.25–7.63; *p* = 0.04), while resection (SIB score 5) was not. **Conclusions:** In our experience, enucleoresection led to a higher risk of PSMs as compared to enucleation. Further studies are needed to assess the differential impacts of resection technique and surgeon’s experience on margin status after robotic PN.

## 1. Introduction 

Current International Guidelines recommend to perform partial nephrectomy (PN) for the treatment of cT1a and cT1b renal tumors whenever technically feasible, as it shows equivalent postoperative oncologic outcomes compared to radical nephrectomy (RN) and has the additional benefit of better renal-function preservation [1,2]. Undeniably, the tumor resection technique employed during PN plays a pivotal role in the amount of preserved healthy renal parenchyma, ultimately influencing postoperative renal function recovery [3,4]. Nevertheless, the question as to whether the type of resection technique performed during PN does affect positive surgical margins (PSMs) rate is still matter of discussion [5,6,7]. Current literature on this issue has been critically influenced by the lack of standardized nomenclature on resection techniques after PN, ultimately leading to a meaningful bias in the assessment of both resection technique performed and estimation of PSMs rate. For this reason, still no consensus exists on the impact of resection technique on surgical margin status after PN, irrespective of the nephrometric characteristics of the tumor, the surgical approach adopted and, not lastly, surgeon’s experience [8,9].

To address this need, a standardized scoring system, the Surface-Intermediate-Base (SIB) Margin score, was first introduced in 2014 to objectively report the resection techniques through a visual assessment of the PN specimen by the surgeon [10], and was recently internally validated by a multicentric study conducted by the SIB Margin Score International Consortium [11]. Due to the increasing use of the robotic approach in the PN worldwide scenario and the non-negligible heterogeneity carried itself by the different resection techniques performed, the assessment of PSMs rate after robotic PN, verified through a standardized tumor-resection reporting system, currently represents a research priority.

As such, we sought to analyze data from our multi-institutional database to address this relevant clinical question. In particular, we conducted this observational study to define the different patterns of resection techniques and to explore their impact on surgical margin status in patients treated with robotic PN for cT1-T2 renal tumors.

## 2. Materials and Methods

### 2.1. Patient and Dataset

After the Institutional Review Board was obtained, data from consecutive patients with cT1-2N0M0 renal masses treated with PN at 16 referral institutions from September 2014 to March 2015 were retrospectively reviewed from a large international multicenter dataset that was prospectively collected (The Surface-Intermediate-Base margin score Consortium). All patients signed a written informed consent before enrolment. For the current study, only patients treated with robotic PN were included. All patients were scored according to the PADUA [12] and RENAL [13] nephrometric classifications, according to the Centre’s preference. The accuracy of tumor complexity stratification by the PADUA and RENAL score was confirmed using a recently developed mathematical converter [14]. Tumor stage was classified according to the 8th edition of TNM criteria [15]. Histopathology was reviewed according to the WHO 2016 classification [16]. The type of resection technique was prospectively assigned in the operatory room on the pathologic specimen according to SIB Margin score and classified as enucleation (SIB score 0–2), enucleoresection (SIB score 3–4) or resection (SIB score 5).

After fixation in a 10% formalin solution, all specimens were step-sectioned at 5-mm intervals, and the entire specimen was analysed. The presence of ink at the resected margins on gross assessment, confirmed by microscopic extension of malignant cells at the stained margins on final histopathological examination, was reported as a PSM. When PSM was confirmed, no re-resection on tumor resection bed was performed.

### 2.2. Objectives of the Study

The objectives of the current study were: (1) to compare the baseline tumor-related characteristics (clinical diameter, cT stage, PADUA score) and operative (surgical approach, operative time, hilar clamping, warm ischemia time [WIT], estimated blood loss [EBL], SIB score), and histopathological (pathological diameter, positive surgical margins, pT stage and tumor histotype) features between patients with positive and negative surgical margins after robotic PN; and (2) to assess independent predictors of PSM among tumor- and surgeon-related factors (including resection technique).

### 2.3. Statistical Analysis

Descriptive statistics were obtained, reporting medians (and interquartile ranges, IQR) for continuous variables and frequencies and proportions for categorical variables, as appropriate. Clinical variables and surgical outcomes between patients with and without PSMs at final histopathological examination were compared using Chi-square for categorical data and the Mann–Whitney U test for continuous variables. Multivariate logistic regression analysis was performed to evaluate clinical and surgical predictors for PSMs, including the following co-variates: tumor diameter (continuous variable), PADUA score (PADUA 6–7 vs. 8–9 vs. ≥10), ischemia (clamp vs. clampless), contact with urinary collecting system (no vs. yes) and SIB score (enucleation vs. enucleoresection vs. resection). Statistical analyses were performed using SPSS v. 27 (IBM SPSS Statistics for Mac, Armonk, NY, USA, IBM Corp). All tests were two-sided with a significance set at *p* < 0.05.

## 3. Results

Overall, 507 patients were enrolled. Among these, 289 met the inclusion criteria for the present study. Median age was 61 (IQR 53–69), while median ASA score and Charlson Comorbidity Index (CCI) were 2 (IQR 2–2) and 1 (IQR 1–2), respectively. Median (IQR) preoperative tumor size for the entire cohort was 3.0 (2.3–4.2) cm and median (IQR) PADUA score was 8 (7–9). After visual assessment of the PN specimen by the surgeon, SIB scores of 0–2 (enucleation), 3–4 (enucleoresection) and 5 (resection) were reported in 53.3%, 27.3% and 19.4% of cases, respectively. Baseline and intraoperative features are summarized in Table 1.

Malignant histology was found in 217 (75.1%) cases and 18 (6.2%) cases of PSM were recorded. PSMs rate was 4.5%, 11.4% and 3.6% in case of enucleation, enucleoresection and resection, respectively. Postoperative and pathological findings are summarized in Table 2.

A comparison between patients with and without PSM is provided in Table 3.

Patients showing PSM had tumors with a higher rate of contact with urinary collecting systems (55.6% vs. 27.3%; *p* < 0.001) and a longer median warm ischemia time (22 vs. 16 min; *p* = 0.02) compared with patients with negative surgical margins. Conversely, no significant differences were found between the two groups in terms of other patient- and tumor-related features. At univariable analysis, pathological diameter (OR: 1.26; 95% CI 1.10–1.44; *p* = 0.001), clampless approach (OR: 1.74; 95% CI 1.14–4.79; *p* = 0.02), contact with urinary system (OR: 1.22; 95% CI 1.11–1.52; *p* = 0.001), PADUA score ≥ 10 (OR: 2.17; 95% CI 1.37–3.44; *p* = 0.001), and enucleoresection (SIB score 3–4) versus enucleation (SIB score 0–2) (OR: 3.11; 95% CI 1.76–5.51; *p* < 0.001) were significantly associated with PSM. With a multivariable model, enucleoresection (SIB score 3–4) versus enucleation (SIB score 0–2) was confirmed as the only independent predictor of PSM at final pathology (OR: 2.68; 95% CI 1.25–7.63; *p* = 0.04), while resection (SIB score 5) was not (*p* = 0.62) (Table 4). A detailed reporting of patterns of resection techinques according to different tumor histotypes, stratified by margin status is provided in Supplementary Table S1.

## 4. Discussion

The complete excision of the renal malignancy is an oncologic goal of paramount significance during PN. Actually, PSMs rate has traditionally been associated with the amount of healthy renal tissue resected with the tumor and consequently with the different resection technique performed. However, whether the type of resection technique does affects the rate of PSMs is still an object of discussion [17]. Recently, two meta-analyses confirmed the oncologic safety of tumor enucleation with regards to PSMs rate as compared to standard PN [5,18]. However, the lack of use of a standardized reporting system led to the inclusion of mixed resection techniques within the tumor enucleation group, thus undermining a truthful comparison. Furthermore, open tumor enucleation was the technique performed in most of the studies, making it arduous to directly translate such evidence to the robotic scenario. By harnessing the SIB score to report resection techniques performed in a standardized fashion, the current manuscript can contribute to the ongoing debate and overcomes the limits of previous studies, ultimately providing novel findings that may help to better contextualize current robotic PN literature.

To our knowledge, the present study represents the largest series so far exploring predictors of PSMs after robot-assisted PN verified through a standardized tumor-resection reporting system. We found that, after adjusting for clinical and pathological variables, enucleoresection, when compared to enucleation, was the only independent predictor of PSMs at final histopathological examination. On the contrary, histopathologic features, clinical tumor characteristics and hilar clamping were not found to influence PSMs rate at multivariable analysis. This finding suggests that, during the phase of the excision, following tumor burdens, such as during enucleation, or resecting a wide parenchymal margin far beyond the tumor contours, such as during resection, may be protective against PSMs as compared to enucleoresection. Most importantly, despite skepticism traditionally raised for tumor enucleation concerning a theoretical higher risk of PSMs, such a result is paramount towards a more comprehensive definition of evidence-based strategies to tailor surgical management in patients eligible for PN. Actually, the rationale for tumor enucleation can be ascribed to the distinct anatomical characteristics of the tumor–parenchyma interface, which allow for the definition of a constant anatomic dissection plane for tumor excision [19]. In particular, the technical feasibility and oncologic safety of tumor enucleation relies upon the presence of a continuous fibrous peritumoral pseudocapsule, which represents a relevant anatomical landmark for the surgeon approaching the renal mass with an enucleative intent. Previous studies highlighted how, while a surgeon performed tumor excision without removal of a macroscopic renal margin, final histopathological examination confirmed the presence of a distinct microscopic layer of healthy renal tissue, which can be ascribed to tumoral pseudocapsule [20]. In this regard, robotic tumor enucleation is not a *zero-margin*, but rather a *microscopic-margin technique*. As such, following the natural cleavage plane between the healthy parenchyma and tumor pseudocapsule and, keeping always in sight the tumor burdens during enucleation, this might contribute to reducing the risk of PSMs. Moreover, by developing the anatomic cleavage plane following tumor pseudocapsule, robotic tumor enucleation might also allow surgeons to widen the indications for PN in case of challenging, highly complex renal masses, especially if they are not perfectly round-shaped or in close contact with the urinary collecting system [21,22]. In experienced hands, robotic tumor enucleation already proved to achieve negative surgical margins in the vast majority of PNs, ultimately providing excellent midterm local control and oncologic outcomes [23,24]. Of note, in our experience, more than half of PN specimens were classified as enucleations (SIB score 0–2) after a visual inspection by the surgeon, confirming a relatively high penetrance of this resection technique among robotic PN surgeons.

Interestingly, clampless PN was not independently associated with a higher risk of PSMs. Some would argue that the type of hilar control approach may theoretically influence the resection technique and surgical margins status, considering the potential advantages of the on-clamp approach in providing less bleeding from the adjacent renal parenchyma during tumor excision, thus guaranteeing a better visualization of the tumor margins. However, a growing body of evidence has recently questioned the hypothetical role of the technique used to manage the renal pedicle in determining either surgical and oncological PN outcomes [25,26,27,28]. Consistent with current literature, in our experience a clampless approach was found to not significantly increase the risk of PSMs as compared to on-clamp procedures in multivariable analysis. Undeniably, the surgeon’s experience also plays a pivotal role in this regard. Nonetheless, this result suggests that a clampless approach, whenever technically feasible, does not undermine the complete excision of the renal malignancy, maximizing functional results at the same time.

Our study is not devoid of limitations. First, PN was performed by experienced surgeons at referral centers. Limited data on surgeon case-volume were available in the SIB database and the learning curve of each surgeon was not considered in the present study. As such, our findings may not be applicable to all surgeon- or center-related scenarios. On the other hand, as all cases were treated robotically between 2014 and 2015, we could not completely rule out that some institutions or surgeons had not yet fully reached the maximum proficiency in their robotic learning curve. Second, histopathological analysis of PN specimens was not centralized. Third, the extent of interobserver variability of SIB score assignment was not evaluated with objective metrics across included centers.

Acknowledging these limitations, the current paper represents the largest series so far to explore the clinical and surgical predictors of PSMs after robot-assisted PN that is verified through a standardized tumor-resection reporting system. In this scenario, we identified future perspectives to improve preoperative surgical planning and maximize both oncological and functional outcomes: (1) three dimensional reconstructions [29,30] can enhance the perceiving of tumor anatomy and vascularization, allowing a wiser and more accurate choice of the most appropriate resection technique; (2) integrating preoperative nomograms [6] and novel radiological scores [31] in a more comprehensive decision-making tool can contribute to tailoring surgical approaches to patients eligible for PN; (3) to accredit only high-volume centers to perform PN might pave the way to the employment of such novel technologies in everyday clinical practice, maximizing postoperative outcomes and cost-effectiveness of PN. However, larger studies are warranted to confirm our results in different clinical scenarios and to assess the impact of resection technique on mid- and long-term functional and oncologic outcomes after PN.

## 5. Conclusions

To our knowledge, this is the first multicenter study using a standardized reporting system to evaluate clinical and surgical predictors of PSMs. In our experience, resection technique was confirmed as the only independent predictor of PSM after robotic PN. In particular, enucleoresection led to a higher risk of PSMs as compared to pure enucleation. Larger prospective studies are needed to validate our findings.

## Figures and Tables

**Table 1:** Preoperative and intraoperative features of 289 patients treated robotic PN for cT1-T2N0M0 renal tumors.

Preoperative Features		
Age, Median (IQR)		61 (53–69)
Gender, n (%)	Male	154 (53.3%)
	Female	135 (46.7%)
BMI, median (IQR)		26.3 (24.0–30.0)
ASA score, median (IQR)		2 (2–2)
ASA score ≥ 3, n (%)		48 (16.6%)
Charlson Score, median (IQR)		1 (1–2)
Tumour diameter (cm), median (IQR)		3.0 (2.3–4.2)
Tumor side, n (%)	Right	154 (53.3)
	Left	135 (46.7)
cT stage, n (%)	T1a	198 (68.5)
	T1b-T2	91 (31.5)
PADUA score, median (IQR)		8 (7–9)
Preoperative eGFR, median (IQR)		87.8 (72.8–97.4)
**Intraoperative features**		
Approach, n. %	Transperitoneal	264 (91.3)
	Retroperitoneal	25 (8.7)
Hilar clamping, n. %		234 (80.9%)
Warm Ischemia Time (min), median (IQR)		16 (14–21)
EBL (cc), median (IQR)		150 (90–210)
Operative time, median (IQR)		140 (115–190)
SIB score, n. %	SIB score 0–2	154 (53.3)
	SIB score 3–4	79 (27.3)
	SIB score 5	56 (19.4)

**Table 2:** Postoperative and histopathological features of 289 patients treated robotic PN for cT1-T2N0M0 renal tumors.

Postoperative and Anathomopathological Features		
Pathological diameter (cm), median (IQR)		3.0 (2.2–4.0)
Tumor nature, n. (%)	Benign	72 (24.9)
	Malignant	217 (75.1)
pT stage, n. (%) (among malignant tumors, n = 217)	pT1a	158 (72.8)
	pT1b	43 (19.8)
	pT2	5 (2.3)
	pT3a	11 (5.1)
Positive Surgical Margins, n. (%)		18 (6.2)
Positive Surgical Margins rate according to resection technique, n. (%)	SIB score 0–2	7/154 (4.5)
	SIB score 3–4	9/79 (11.4)
	SIB score 5	2/56 (3.6)
RCC Histotype, n. (%)	Clear Cell (cc-RCC)	149 (51.6)
	Papillary (p-RCC)	34 (11.8)
	Chromofobe (ch-RCC)	22 (7.6)
	Oncocytoma	46 (15.9)
	Angiomyolipoma	26 (9.0)
	Other RCC subtypes	12 (4.2)

**Table 3:** Comparison of clinical, surgical and postoperative features according to surgical margin status in 289 patients treated with robotic PN for cT1-2N0M0 renal tumors.

Preoperative Features		NSM (n = 271)	PSM (n = 18)	*p* Value
Age, median (IQR)		61 (54–69)	64 (52–68)	0.99
Gender, n (%)	Male	170 (62.7)	13 (72.2)	0.41
	Female	101 (37.3)	5 (27.8)	
BMI, median (IQR)		26.3 (24–30)	25.8 (23.7–32.6)	0.19
ASA score, median (IQR)		2 (2–2)	2 (2–3)	0.26
ASA score ≥ 3, n (%)		43 (15.9)	5 (27.8%)	0.20
Charlson Score, median (IQR)		1 (1–2)	1 (1–2)	0.43
Tumour diameter, median (IQR)		3.0 (2.3–4.2)	2.8 (1.9–3.9)	0.42
cT stage, n (%)	T1a	184	14	0.38
	T1b-T2	87	4	
PADUA score, median (IQR)		8 (7–9)	8 (7–9)	0.31
Contact with urinary collecting system, n (%)		74 (27.3)	10 (55.6)	<0.001
Preoperative eGFR, median (IQR)		87.8 (73.2–98.2)	86.5 (67.4–93.5)	0.39
**Intraoperative Features**		**NSN (n = 271)**	**PSM (n = 18)**	***p* Value**
Approach, n. %	Transperitoneal	246	18	0.18
	Retroperitoneal	25	0	
Hilar clamping, n. %		217 (80.1)	16 (88.9)	0.37
Warm Ischemia Time (min), median IQR		16 (13–20)	22 (16–26)	0.02
EBL (cc), median IQR		150 (90–210)	160 (95–220)	0.63
Operative Time, median IQR		140 (115–190)	145 (120–200)	0.58
SIB score, n. %	SIB score 0–2	147 (54.2)	7 (38.9)	0.08
	SIB score 3–4	70 (25.8)	9 (50)	
	SIB score 5	54 (19.9)	2 (11.1)	
**Postoperative Features**		**NSN (n = 271)**	**PSM (n = 18)**	***p* Value**
Pathological diameter (cm), median (IQR)		3.0 (2.2–4.0)	2.9 (1.8–4.0)	0.74
Tumor nature, n. (%)	Bening	70 (25.8)	2 (11.1)	0.08
	Malignant	201 (74.2)	16 (88.9)	
pT stage, n. (%) (among malignant tumors, n = 217)	pT1a	146 (72.6)	12 (75)	0.47
	pT1b	41 (20.4)	2 (12.5)	
	pT2	5 (2.5)	0 (0.0)	
	pT3a	9 (4.5)	2 (12.5)	
RCC Histotype, n. (%)	Clear Cell (cc–RCC)	137 (50.6)	12 (66.7)	0.59
	Papillary (p–RCC)	31 (11.4)	3 (16.7)	
	Chromophobe (ch–RCC)	21 (7.7)	1 (5.6)	
	Oncocytoma	45 (16.6)	1 (5.6)	
	Angiomyolipoma	25 (9.2)	1 (5.6)	
	Other RCC subtypes	12 (4.4)	0 (0.0)	

**Table 4:** Univariable and multivariable analysis analyzing the predictive factors for surgical predictors of PSMs.

z		Univariable Analysis			Multivariable Analysis			
		OR	95% CI	*p* Value	Standard Error	OR	95% CI	*p* Value
Pathological diameter (continuous)		1.26	1.10–1.44	0.001	0.22	0.81	0.54–1.25	0.36
Ischemia	Clampless	1.74	1.14–4.79	0.02	0.79	0.55	0.12–2.58	0.45
	Clamp (ref)	-	-	-	-	-	-	-
Contact with urinary collecting system	Yes	1.22	1.11–1.52	0.001	0.74	1.33	0.78–2.49	0.21
	No (ref)	-	-	-	-	-	-	-
PADUA score	≥10	2.17	1.37–3.44	0.001	0.78	0.74	0.16–3.41	0.69
	8–9	1.46	0.88–2.41	0.14	0.57	1.53	0.49–4.67	0.45
	6–7 (ref)	-	-	-	-	-	-	-
SIB score	5	0.92	0.83–1.01	0.09	0.84	1.56	0.30–8.09	0.62
	3–4	3.11	1.76–5.51	<0.001	0.54	2.68	1.25–7.63	0.04
	0–2 (ref)	-	-	-	-	-	-	-

## Data Availability

Data available on request due to restrictions e.g., privacy or ethical.

## Supplementary Materials

The following supporting information can be downloaded at: https://www.mdpi.com/article/10.3390/jcm11071765/s1, Table S1: Analysis of the patterns of resection techniques according to different tumor histotypes, stratified by margin status.
