# Supplementary material for: Predictors of Positive Surgical Margins after Robot-Assisted Partial Nephrectomy for Localized Renal Tumors: Insights from a Large Multicenter International Prospective Observational Project (The Surface-Intermediate-Base Margin Score Consortium)
Source: J Clin Med. 2022 Mar 23;11(7):1765. doi: 10.3390/jcm11071765 (PMC8999836; doi:10.3390/jcm11071765)
Supplement: Supplementary file 1 [file jcm-11-01765-s001.zip › jcm-1543312-supplementary.pdf]

Supplementary Table S1: Analysis of the patterns of resection techniques according to different tumor histotypes, stratified by margin status.

		Positive Surgical Margins	
		No (n=271)	Yes (n=18)
**Enucleation** (SIB 0-2) (n=154) (n,%)	ccRCC	76 (51.7)	4 (57.1)
	pRCC	17 (11.6)	0 (0)
	chRCC	11 (7.5)	1 (14.3)
	Onco	26 (17.7)	1 (14.3)
	AML	9 (6.1)	1 (14.3)
	Other	8 (5.4)	0 (0)
**Enucleoresection** (SIB 3-4) (n=79) (n,%)	ccRCC	34 (48.6)	6 (66.7)
	pRCC	11 (15.7)	3 (33.3)
	chRCC	5 (7.1)	0 (0)
	Onco	12 (17.1)	0 (0)
	AML	6 (8.6)	0 (0)
	Other	2 (2.9)	0 (0)
**Resection (**SIB 5) (n=56) (n,%)	ccRCC	27 (50.0)	2 (100)
	pRCC	3 (5.6)	0 (0)
	chRCC	5 (9.3)	0 (0)
	Onco	7 (13.0)	0 (0)
	AML	10 (18.5)	0 (0)
	Other	2 (3.7)	0 (0)
